# Healthcare providers' views and perceptions on post-mortem procedures for cause of death determination in Southern Mozambique

**DOI:** 10.1371/journal.pone.0200058

**Published:** 2018-07-06

**Authors:** Maria Maixenchs, Rui Anselmo, Ariadna Sanz, Paola Castillo, Eusebio Macete, Carla Carrilho, Jaume Ordi, Clara Menendez, Quique Bassat, Khatia Munguambe

**Affiliations:** 1 ISGlobal, Hospital Clinic—Universitat de Barcelona, Barcelona, Spain; 2 Centro de Investigação em Saúde de Manhiça, Maputo, Mozambique; 3 Department of Pathology, Hospital Clinic of Barcelona, Universitat de Barcelona, Barcelona, Spain; 4 National Directorate of Public Health, Ministry of Health, Maputo, Mozambique; 5 Department of Pathology, Maputo Central Hospital, Maputo, Mozambique; 6 Faculty of Medicine, Eduardo Mondlane University, Maputo, Mozambique; 7 Consorcio de Investigación Biomédica en Red de Epidemiología y Salud Pública (CIBERESP), Madrid, Spain; 8 Catalan Institution for Research and Advanced Studies (ICREA), Barcelona, Spain; 9 Pediatric Infectious Diseases Unit, Pediatrics Department, Hospital Sant Joan de Déu (University of Barcelona), Barcelona, Spain; University of Manchester, UNITED KINGDOM

## Abstract

**Background:**

The minimally invasive autopsy (MIA) is being investigated as an alternative to the complete diagnostic autopsy (CDA), gold standard for CoD determination, in settings where CDA is unfeasible and/or unacceptable. We aimed to explore healthcare providers’ views and perceptions on theoretical and factual acceptability of the CDA and the MIA.

**Methods:**

A qualitative study, combining ethnographic and grounded-theory approaches, was conducted within a project aiming to validate the MIA tool against the CDA for CoD investigation. We present data on in-depth and semi-structured interviews of 33 healthcare providers operating within the formal and informal health services in Southern Mozambique. MIA perception was analysed through the theory of diffusion of innovations.

**Results:**

All participants considered CDA useful for CoD determination. CDA was perceived reliable, but the unpleasant nature of the procedure and its associated infection risk were the main perceived disadvantages. Participants considered the MIA simple, easy and quick to perform; likely to meet families’ expectations to know the CoD, and able to provide evidence-based knowledge for disease management. Concerns were raised on its reliability compared to the CDA. Family's emotional status and accessibility to decision-makers were mentioned as principal barriers for MIA performance. The main jeopardizing factors for MIA implementation were the shortage of required resources and the significant proportion of people dying at home. Key facilitators for MIA acceptance included the need for the support from community and religious leaders, provision of clear information to the community, and accompaniment to bereaved families.

**Conclusions:**

Healthcare providers consider the MIAs potentially more acceptable and feasible than CDAs in places where the latter have shown significant implementation challenges. A clear understanding of healthcare provider’s perceived barriers and facilitators for conducting post-mortem procedures in general, and MIAs in particular, will shed light on their future field implementation for more robust mortality surveillance.

## Introduction

In low and middle-income countries (LMIC), significant challenges exist in order to account for and establish what the main causes of death at the individual and population level are. Although much stronger recommendations are now in place to strengthen vital registration systems in these settings, the reality is that in many rural areas of the poorest countries, a considerable number of births and deaths are not captured by the national registration platforms. In sub-Saharan Africa, regional death certificate coverage may be as low as 10%, and the quality of cause of death data remains poor, essentially relying on verbal autopsy and clinical data [1]. Mozambique, a paradigmatic example of a sub-Saharan African country is no exception to this, whereby mortality data are scarce, incomplete and often unreliable [2].

The minimally invasive autopsy (MIA) has been validated as a strong alternative for cause of death determination (CoD) in LMIC where the gold standard method, the complete diagnostic autopsy (CDA), is unfeasible to implement [3–6]. In such countries, CDA can only be performed in top-level health facilities, whereas in the community verbal autopsies are the commonly used method for CoD determination. However, VA has important limitations and poor specificity [7].

The MIA consists of a series of post-mortem punctures using biopsy needles aiming to obtain tissue samples and body fluids within the first hours after death, which are then submitted for a thorough histopathological and microbiological investigation of the underlying CoD [8]. The validation of this new MIA protocol for CoD investigation in LMIC has been conducted in Mozambique, in a head-to-head comparison against the gold standard methodology, the CDA. The results have shown, in the different age groups studied, including adults, children, neonates, stillbirths and maternal deaths, a moderate to substantial concordance between the two methods (Kappa values of the concordance analyses in the different age groups ranging from 0.40 to 0.78) [3–6]. Therefore, the MIA can potentially act as an alternative for the CDA in those areas where the latter cannot be easily conducted, provided that the procedure is accepted not only by the community but also by healthcare providers.

Healthcare providers are regularly in contact with the event of death and are often responsible for the interaction with the relatives of dying or dead individuals (including grief counselling and consent for post-mortem procedures). Additionally, some of these health professionals may be potential candidates to perform the MIA in the future. Previous qualitative studies about health providers’ perceptions and attitudes related to post-mortem methods and communication with beavered families (including informed consent) are mainly focused on High-Income Countries (HIC) [9–12].

In this analysis we aimed to explore local healthcare providers' perceptions and attitudes regarding post-mortem methods in general, and MIAs in particular, and the perceived advantages, disadvantages, barriers and facilitators of this technique in the context of low resourced settings. The theory of diffusion of innovations (DOI) introduced in 1962 by Rogers [13] is useful to understand why and how rapidly new tools and interventions are adopted (or not), despite the evidence of their potential benefits [14]. The DOI focuses on the multiple factors that can affect any implementation, taking into consideration the innovation itself, the communication channels and the decision-making processes, time and the social systems [13].

## Methods

### Study site and population

The study took place in Maputo city and in the District of Manhiça, both in Southern Mozambique. Maputo, the capital of Mozambique, is a city with a population of approximately 2 million people, most of them living in peri-urban areas. The city is served by the Maputo Central Hospital (HCM), where CDAs are routinely conducted, as well as three general hospitals and several health facilities. Besides conducting routine CDAs, from November 2013 to March 2015, the HCM hosted a MIA validation study, in which 282 MIAs were performed on adults (including maternal deaths), children, neonates and stillborn deceased at the hospital, and compared against CDAs as the gold standard [3–6].

Manhiça District is a rural area, located 80 Km north of Maputo City, with a population of 183,000 inhabitants covered by a Health and Demographic Surveillance System (HDSS) ran by the Manhiça Health Research Centre, previously described elsewhere [8,15]. Health services in Manhiça District are provided by a district hospital, a rural hospital, and 12 primary-level health centers, none with the capacity to conduct post-mortem procedures at the time when the current study was conducted [8].

Formal and informal health system structures coexist both in Maputo and in Manhiça. Informal healthcare providers include, among others, traditional healers, herbalists, traditional birth attendants, informal drug providers and knowledgeable elders. The target population of this specific analysis included healthcare providers from both geographical areas, defined as those regularly in contact with death events, both within the formal and informal health system, and involved or not in post-mortem procedures (including the performance of MIAs). This included medical doctors, nurses, midwives, community health workers (CHW), traditional birth attendants (TBA) and traditional healers.

### Study design

This article reports findings from a qualitative study conducted in the context of a larger multicentre study [Validation of the Minimally Invasive Autopsy tool for cause of death investigation in developing countries (CaDMIA)], aiming to assess the performance of a minimally invasive autopsy tool (MIA) in comparison to the CDA [3–6]. The overall qualitative component, conducted in 5 countries (Gabon, Mali, Kenya, Mozambique, Pakistan), was based on a combination of ethnographic [16] and grounded theory [17,18] approaches to data collection and analysis. The aim was to understand the phenomenon of death and post-mortem methods from the local communities perspectives (including healthcare providers), and to contribute to theory based on data systematically obtained from social research [17,18]. In consequence, there were no predetermined specific hypotheses, but broad research questions focused on the area of interest. The research questions addressed by this specific study, focussed only in Mozambican healthcare providers, included: i) How do healthcare providers perceive CDAs and MIAs? ii) What are the barriers, facilitators and requirements for MIA implementation according to healthcare providers in Southern Mozambique?

### Sampling

A minimum sample size of 30 participants was defined based on similar studies' experience in reaching saturation (the non-addition of new concepts after the continuous recruitment of more interviewees) [19,20]. Purposive sampling was used to only enrol those who were regularly in contact with death (particularly corpses and bereaved family members), specifically at the time of death and/or shortly after the death event. Those included were formal healthcare providers (medical doctors, nurses, clinical officers, midwifes, health facility administrative personnel, staff from the health facility morgues and community health workers), and informal healthcare providers (traditional birth attendants and traditional healers). Of note, some of the interviewed healthcare providers in Maputo had actually witnessed CDAs and MIAs being conducted, while those interviewed in Manhiça had generally never seen post-mortem techniques being conducted and thus, their perceptions were discussed from a theoretical point of view.

### Recruitment strategy

Formal healthcare providers were identified and recruited at health facilities by the study social scientists and research assistants. Informal healthcare providers were identified and recruited by research assistants through a series of community meetings that were held to explain the CaDMIA ethnographic study within the study area [8].

### Data collection

Thirty-three semi-structured interviews were conducted with healthcare providers (in rural Manhiça and urban Maputo) between November 2013 and February 2015. Data were collected by social scientists and local research assistants based on a guide of open questions linked to the main research questions, but always focusing on the respondents ‘train of thoughts”, allowing emerging themes to be captured. Interviews were conducted in Shangaan or Portuguese, according to participant's preference, were audio recorded (when permission was given by participants) and transcribed. Detailed field notes were taken when no permission was given for recording.

### Analysis

While the overall qualitative study used grounded theory, data from this specific component were thematically coded using Nvivo 10 (QSR International Pty Ltd). This coding process entails identifying passages of text related to the topics of interest to the research question and labelling them with specific themes and sub-themes. As transcripts were gradually imported into the Nvivo project and thoroughly read, codes were refined, added or eliminated, depending on their relevance to the main research question, the already coded data, and the emergent data. By grouping together similarly coded text, themes were constructed, discussions were built around each theme and around links between themes and subthemes, and from these discussions, the main conclusions emerged [21].

The theory of diffusion of innovations (DOI) [13], was used to asses MIAs perceptions from the stand point of an innovation. Diffusion of innovations is the process by which a new idea, practice, or object that is perceived as new, is communicated through certain channels over time among the members of a social system. The characteristics of innovations, as perceived by individuals, help to explain their different rate of adoption. The analysis focuses on the 5 characteristics or elements, described in the theory, namely i) relative advantage; ii) compatibility; iii) complexity; iv) trialability; and v) observability [13].

This study complies with the Consolidated Criteria for Reporting Qualitative Research (COREQ) checklist [22].

### Ethical considerations

The study was approved by the Manhiça Health Research Centre's Institutional Review Board (CIBS-CISM) and the Mozambican National Bioethics Committee (Ref. 342/CNBS/13) in Mozambique, and by the Ethics Committee of Barcelona’s Hospital Clínic (File 2013/8676) in Spain. Written informed consent was obtained from all participants. In the case of illiteracy, participants provided a thumbprint that was countersigned by an impartial witness, guaranteeing that participation had been informed and voluntary. All data were managed based on unique identification numbers so as to guarantee the respondent’s anonymity.

## Results

A total of 33 respondents were interviewed, 39% (13/33) in Maputo and 61% (20/33) in the Manhiça study area. Fifty-eight per cent (19/33) of all participants were males, 67% (22/33) had completed professional training or further studies, and the majority (79%; 26/33) was Christian. Detailed socio-demographic characteristics of the study participants are shown in Table 1. All whom were approached by the study team accepted to participate.

**Table 1:** Socio-demographic characteristics of participants and experience with complete diagnostic autopsies and minimally invasive autopsies.

Participants	Maputo City n (%)	Manhiça District n (%)	TOTAL N (%)
**Gender**			
Male	9 (69)	10 (50)	19 (58)
Female	4 (31)	10 (50)	14 (42)
**Age group, years**			
18–29	5 (38)	2 (10)	7 (21)
30–49	7 (54)	10 (50)	17 (52)
>50	1 (8)	8 (40)	9 (27)
**Education**			
No schooling	0 (0)	0 (0)	0 (0)
Primary^1^	3 (23)	8 (40)	11 (33)
Secondary	0 (0)	0 (0)	0 (0)
Professional Training	2 (15)	11 (55)	13 (40)
University or higher	8 (62)	1 (5)	9 (27)
**Occupation**			
Medical Doctor	6 (46)	1 (5)	7 (22)
Clinical officer	2 (15)	4 (20)	6 (18)
Nurse	0 (0)	3 (15)	3 (9)
Midwife	0 (0)	2 (10)	2 (6)
Pathology assistant	2 (8)	0 (0)	1 (3)
Hospital Registrar	0 (0)	1 (5)	1 (3)
Mortuary assistant	3 (23)	1 (5)	4 (12)
Community health worker	0 (0)	2 (10)	2 (6)
Traditional birth attendant	0 (0)	3 (15)	3 (9)
Traditional healer	0 (0)	3 (15)	3 (9)
**Religion**			
Christian^2^	9 (69)	17 (85)	26 (79)
Muslim	1 (8)	1 (5)	2 (6)
Animist	0 (0)	2 (10)	2 (6)
Atheist	1 (8)	0 (0)	1 (3)
Unknown	2 (15)	0 (0)	2 (6)
**Experience with post mortem procedures**			
Has conducted/seen CDA			
Yes	12 (92)	7 (35)	19 (56)
No	1 (8)	13 (65)	14 (42)
Has conducted/seen MIA			
Yes	6 (46)	0 (0)	6 (18)
No	7 (54)	20 (100)	27 (82)
**TOTAL**	**13 (39)**	**20 (61)**	**33 (100)**

Values are n (percent) unless indicated otherwise

^1^ Includes three participants with incomplete primary studies

^2^ Includes Catholic, Protestant or Evangelist, or Christian undetermined.

### Views and perceptions of healthcare providers regarding Complete Diagnostic Autopsies (CDA)

Twelve of the participants from Maputo (12/13) had been involved in the performance of at least one CDA. All but two (11/13) were working at the Maputo Central Hospital, of which six (6/11) conducted CDAs as part of their routine activities. No CDAs were performed in Manhiça, where less than half of the participants (7/20) reported any previous participation in post-mortem methods, always in the context of their professional training.

Participants from Maputo explained that the CDA is a procedure consisting in opening the body, conducting a visual examination of the organs, collecting samples for histology and, finally, stitching the incisions. The description also included details on the nature and localization of the incisions, the main organs observed, and the material used.

Among formal healthcare providers from the Manhiça area, the CDA was defined as a technique consisting in opening the body, exploring the organs and obtaining samples for analysis. Neither the three CHWs nor the informal providers (three TBA and three traditional healers) were able to explain the procedure. A traditional healer referred to the CDA as *the opening of the head*.

Some participants explained that the CDA was not easy to perform, unpleasant, and caused physical and mental discomfort to the staff performing it. Additionally, it was said that specialized training was needed to conduct a CDAs and that the staff in charge had to be psychologically prepared to face the process. The strong smell, the large incisions, the exposure to the organs and the blood were other mentioned disturbing issues.

Participants also expressed concerns regarding returning a significantly disfigured body to the families, and its impact on their emotions, aggravating the existing shock from the death of a beloved one, and causing additional stress. Health providers reported the discomfort related to requesting consent or informing the families about CDAs, particularly if influenced by rumors about the procedure, although families normally were open to receiving information about the method and the reasons for requesting the CDA.

*“They just have heard of it* (CDAs) *from others*. *That’s why there are some people who reject the autopsy* (CDA) *but they do not know really what it is*, *they think autopsy* (CDA) *is to take organs away to be stored for studying them*. *It is as they were taking home an empty body*.” Medical doctor, 36 years old, female

One of the major concerns regarding CDAs among health professionals with experience of CDA performance was the risk of infection.

*“There* (when doing autopsies) *the risk is* … *Sometimes*, *well*, *most of the time*, *we work with seropositive* (HIV positive) *bodies*, *so one can never be too careful*.*”* Pathology assistant, 27 years old, male

Despite the abovementioned concerns, the majority of formally trained health professionals considered that the CDA was a useful tool for getting to know the real CoD, especially when it was not clear, and that learning from the results could help improving disease management and diagnosis in the future.

*“[CDA] is a way we have to clarify specific causes that*, *when the person was alive*, *we were not able to identify*. *(…) On the other hand*, *we may have thought we were in front of a certain disease and when we have the results from histology*, *from the autopsy*, *it reveals other things*, *and so we end up learning*. *Yes*, *we end up learning*.” Medical doctor, 35 years old, female.

*“The first time I saw (a CDA) it was really shocking*. *But after this*, *I realized it was necessary to know the real cause of death*. *We saw ischemia cases*. *Those things go unnoticed*, *you just discover them when the heart is directly observed and you can see the changes occurring in this organ*.*”* Medical doctor, 33 years old, female

However, health professionals were also aware that postmortem examinations could elucidate the real underlying CoD, potentially questioning the clinician’s diagnostic and prognostic capacity.

*“I am afraid people will say “no” [to CDA]*, *because when you explain to the family that you want to do the autopsy*, *that you want to confirm*, *to discover the cause of death* … *the patients*, *when they come to you*, *they expect you to know*. *So*, *convey to them the idea that “I do not know”*, *“we do not know” is difficult*.*”* Medical doctor, 38 years old, female.

Finally, some participants explained that health professionals’ negative connotations to CDAs should be demystified through education and information. Working at the pathology department or at the mortuary of the Maputo Central Hospital involved stigmatization, according to some participants, particularly for assistants and mortuary personnel. One of them revealed that pathology department and mortuary staff members were uncomfortable with disclosing their profession or details of workplace (i.e., the pathology department or the mortuary services) because, otherwise, they would be perceived as *crazy or drug addicts or even as bad persons*. The same participant mentioned that, even colleagues from the same hospital might treat them differently when finding out they were working in this area.

### Views and perceptions of healthcare providers regarding Minimally Invasive autopsies (MIA)

Six participants interviewed in Maputo (6/13) were involved in conducting MIAs as part of their routine activities. All of them were also familiar with CDAs. At the Manhiça area, no participant had ever seen or participated in a MIA.

The MIA was viewed by all participants, including those without previous experience with the procedure, as a *simple*, *easy* and *quick* technique that *anyone* could perform after appropriate training. In their opinion, the MIAs could be performed anywhere, even in the absence of formal pathology facilities, or at the household level.

*“We (health professionals) need something quick*, *that does not take us a lot of time*. *So*, *it is preferable we do MIA (than CDA)*, *after an appropriate training*.*”* Clinical officer, 33 years old, male.

Participants who had previously conducted a MIA, considered that the method was cheaper, less unpleasant and less time consuming compared to CDAs, with a duration of about 30–45 minutes, as opposed to the ~2 hours required for CDAs. Concerns did however arise regarding the cost of the needles and additional laboratory tests.

The main advantage for those performing MIA was that the technique did not involve major incisions: on one hand, this allowed the procedure to be *clean*, there was no blood and *mess*, there was *less disgusting smell*, and the risk of infection (although it remains a serious concern) was believed to be significantly lower. On the other hand, returning an apparently intact body would remove additional stress to the family, allow the body to be shown during the ceremonies and avoid suspicions regarding organs removal.

“*With MIA*, *you manage to do a kind of autopsy that people at home might not notice*. *I mean*, *everything is fine*. *Because the way the autopsy (CDA) is done…*. *It is not possible to open the coffin and stay there*, *seeing the deceased*, *that way… the head… all… I mean*, *people end up crying twice*. *With MIA*, *people do not notice that a study has been done and you might reach a diagnosis as well*.*”* Pathology assistant, 38 years old, male

A disadvantage highlighted by interviewees of the MIA in comparison to the CDA was a potentially reduced performance of the tool in terms of reaching a diagnosis, as the procedure consists just on the analysis of small samples and does not allow an open-eyed macroscopic analysis.

*“A disadvantage (of MIA) is that you cannot visualize the whole organ*. *Because to reach a diagnosis*, *in some cases*, *just for the mere visualization of the lesions*, *we reach the diagnosis*.*”* Medical doctor, 26 years old, male.

### Facilitators, barriers and requirements for MIA implementation from healthcare providers ‘perspective

The interest in knowing the CoD was mentioned by almost all participants as a facilitator for MIAs to be performed in the future. According to healthcare providers, families would clearly want to know or clarify the CoD of their deceased relatives. To discover the CoD through the MIA would allow taking measures in case of contagious or *family* diseases. Further, the mere fact *of knowing* the cause of death would remove uncertainties about the circumstances of the death, including the avoidance of witchcraft accusations, provision of peace, and release from distress among family members. The nature of the technique, not implying large cuts as compared to the CDA, would avoid rumors about organ stealing or trafficking as well as it would minimize the visual shock of the dead body.

*“Families would accept MIA*. *They will be reassured about the cause of death*. *For example*, *what happens* (is that) *our brothers come back from the border*, *from South Africa*, *Swaziland… People do not know which conditions*, *what kind of life he/she was leading*. (He/She) a*rrives here* (home) *ill*, *and dies*.” Nurse, 38 years old, male.

*“This (MIA) is important*, *because there is no discussion about someone being killed*. *The person died because of an ordinary disease*.*”* CHW, 48 years old, female

Besides the family, healthcare providers, both within the formal and the informal health system, also revealed their personal interest in knowing the CoD of their patients and their communities. The method was considered as a contribution to science because it may provide better knowledge about diseases, assist on future diagnoses and treatments, and save lives. Additionally, a midwife from rural Manhiça mentioned that MIAs would be a good tool for evaluating health professional's performance and *quality control* at the health facilities.

*“H/She (the deceased) has TB (tuberculosis)*, *HIV associated… all… in an advanced stage*. *Why to perform a MIA*? *You have no need*. *Just in the case of suspecting… épa*!*… not knowing the cause*, *isn't it*? *Or maybe because of doctor's carelessness*, *I don’t know*. *Then*, *yes*. *It could be a drug poisoning; they gave the wrong medicine*. *Yes*. *In this case*, *yes”*. Midwife, 34 years old, female

Participants with higher level of formal education reported more concerns about the margin of error of the MIA tool than the other participants. Some doctors stressed that the technique had not been validated yet, therefore for them it was too soon to accept its reliability.

*“Quite frankly* … *for infectious diseases maybe we could have reliable results* (with the MIA), *but for non-infectious diseases I don’t think so*. *The consistent one there is “the classic one”* (CDA) *because we are seeing the organ and we are sure… we are there*! *Just to collect a sample… I don’t know if this is so reliable*.*”* Medical doctor, 27 years old, female

One of the barriers for MIA implementation reported by informal healthcare providers and those working in isolated areas of Manhiça District was related to the fact that many people die at home and are buried in the community, in the absence of any contact with the health system. This “invisibility at death” [23] is common for all age groups, but even more for stillbirths and early neonatal deaths, for whom the burial rituals are often kept secret, and performed by just a couple of respected elder women of the community, at night or dawn, making the tracking of those deaths even more complex.

Participant (P): “If he/she dies at home … There is no other way, we have to wait. When 24 hours has passed, he/she is buried. There is nothing else to do.”

Interviewer (I): “Don't you bring him/her to the hospital (morgue)?”

P- “No, we don’t bring him/her to the hospital”

I–“And when is hot, the body did not get damaged?”

*P- “Yes*. *That’s why*, *if 24 hours have passed*, *he/she is buried*. *Sometimes*, *if the person dies at night*, *when there are no relatives coming from Maputo or from far away*, *at the end of the day he/she can be buried*.*”* CHW, female, 42 years old

“*When giving birth and the baby turns backwards* (dies) *we just bury him/her*. *The way we bury him/her… It is not the same as an adult*, *you do not need a coffin*, *it can be just a basin*. (…) *We look for a shady place*, *under the trees*, *a place that is fresh*, *is dark* (…) *because that child does not deserve to burn by the sun*.*”* TBA, female, 64 years old

Almost all participants reported that the MIA *per se* might not clash with traditional beliefs. Conversely, few participants explained that MIA could conflict with religious beliefs, specifically with Islam. The fact that Muslims must bury the body as soon as possible, preferably within the first 24 hours after the death, could jeopardize acceptance of the procedure from the Muslim community. Additionally, the belief that the death was *God's will*, according to most religions, could also compromise acceptance of MIAs. Some healthcare providers also expressed this fatalistic notion of death, as a few reported that the information obtained through the MIA would not offer any additional value since the person was already dead.

Poor access to family’s decision makers by the health personnel asking for consent to MIA could constitute a barrier. A few participants reported that the decision makers were often not available immediately after the death, as they stay at home to receive condolences or to take care of funeral arrangements. Moreover, some participants made clear that if the MIA implied delays in funerals and ceremonies, family members would be reluctant to accept the procedure.

Family state of mind around the death and family emotions were raised by participants, both from Maputo and Manhiça areas, as an important concern regarding MIA acceptance. Some health professionals considered it may be challenging to approach family members, to discuss and explain the MIA and to ask for consent in such an early and acute moment of pain and grief. Some healthcare providers, mainly from the informal health system, stressed the need for health personnel conducting MIAs to be kind, sensible and empathic with the family of the deceased, and open to respond to any doubt at any time. In alignment to this issue, a participant in Maputo, who was familiar with MIAs, stated that respect was crucial; emphasizing that professional's actions should stay true to what is promised to the family.

*“You must comply with what is said*. *You cannot say to somebody you are going to hand the body at 1pm and they do not receive the body at 1pm*. *Or that the body will have 3 punctures and at the end there are 5*. *When dealing with patients*, *they trust you (…) If you say you are a doctor*, *they trust you*. *They trust you*. *I would trust*!*”* Medical doctor, 33 years old, female

Participants from both settings felt that the health system may not be prepared for the MIA implementation, on account of potential shortages of material and human resources, and inadequacy of equipment. Personnel from the primary healthcare centres reported that deaths at their facilities were rare, as all the patients with a severe or life-threatening diagnosis were referred to higher-level facilities, limiting the utility of conducting MIAs at the primary health centres.

In terms of requirements for the MIAs to be embraced by the health system and the communities, all participants reported that information and communication were crucial for future MIA implementation. This information should have to be clear, detailed and in an understandable language, and should be delivered to the family, the community and the community and religious leaders. This information, according to participants, should include that MIA's purpose is to confirm or discover the CoD, and the characteristics of the technique. Support from local community leaders would be key to implement the MIA. In terms of what to communicate regarding the nature of the method, a participant from rural Manhiça stressed that a key message to families should be that the MIA does not require *to open the body*, as some relatives believed that CDAs are disrespectful to the deceased. Post-procedural feedback to the families regarding the CoD after results become available would also facilitate the acceptance of the technique.

*“The first thing that has to be done is to gather the community and to inform that the hospital wants to work in collaboration with them in relation to death*, *because the hospital wants to know about the disease that killed someone*. (…) *People don't like when someone suddenly shows up and starts doing things*, *without being previously informed*. *But when the community has been informed and knows*, *and if you remind them again when it starts*, *people are not difficult*.*”* CHW, 56 years old, female

Regarding the community level, health professionals from the Manhiça area widely mentioned support from community and religious leaders as crucial for a successful future MIA implementation in the context of CoD investigation. They mentioned that local leaders at the level of the neighborhood (known as *Secretários de Bairro)* were the decision makers, together with the heads of households. In Maputo, the collaboration between the Ministry of Health, the Association of Traditional Healers of *Mozambique* (AMETRAMO) and people performing the funeral ceremonies was seen as essential. The approval of the Ministry of Health, together with the consideration of MIA as a compulsory procedure for all deaths were particularly mentioned by mortuary personnel from Maputo as possible facilitators.

Participants, both from Manhiça and Maputo study areas, also mentioned incentives as possible facilitators for MIAs implementation, and in particular 1) Provision of free transport or transport support for the corpse after the MIA has been concluded; 2) Financial incentives for the family (such as money or the provision of the coffin); and 3) Performing MIAs free of charge.

*“Today*, *there are educated people… those ones*, *are the ones that ask for autopsies*. *Something happens*, *they want to know… those are the ones that prefer even to pay to have the cause of death*. *So*, *if we can have that* (MIAs) *for free*, it *will be an advantage”*. *Pathology assistant*, 27 years old, male

## Discussion

Community acceptability of the CoD investigation methods currently employed in many LMICs, has rarely been a matter of concern, as, for the case of the verbal autopsy, no direct contact with the deceased is required, or, for the case of pre-mortem clinical records and/or death certification registries, the necessary contact can be seen as a continuation of the already established interaction during the clinical illness, within the well-accepted normal routine clinical care. However, implementation of any post-mortem method—irrespective of its nature—to help refine the estimation of CoD in areas where such approaches have been seldom utilized, requires a profound prior understanding of what is locally acceptable and feasible [8]. The healthcare providers' perspective on post-mortem procedures is critical, because they are regularly in contact with death; they interact with the relatives of deceased persons; and they are potential candidates to perform these procedures. This study successfully explored healthcare providers’ perspectives on the practice and use of post-mortem methods (CDA and MIA) for CoD determination, as well as identified their perceived barriers, facilitators and requirements of MIA implementation, to better understand implications for their future use for CoD investigation.

Most of the findings regarding views and perceptions of both the CDAs and the MIAs among healthcare professionals are consistent. Indeed, the most reported perceived advantage of both tools was the provision of information for CoD determination to families and health professionals. Confirmation of the CoD through a reliable methodology appears to give peace and knowledge to families and health professionals alike. Health professionals, however, may either feel reassured by this knowledge, using it as a motivational driver to improve their performance, or contrarily feel threatened by its auditing potential. It is therefore critical to engage them from the very beginning of the implementation of any post-mortem methodology to ensure that this critical professional group becomes its major supporter, rather than its principal opponent.

As in previous studies, MIA’s reliability in terms of reaching a diagnosis was perceived as lower than that of the CDA [10,24]. The risk of infection, although mentioned by health professionals for both methods, was perceived to be lower for the MIA as this technique is less invasive, similarly to what was found in previous studies [24].

Both post-mortem methods require interaction with mourning families, a factor identified in previous studies as a potential barrier for acceptability and feasibility [11,24–28]. In this study we confirmed that the few health providers who conduct CDAs as part of their routine still regard negatively the effect that the CDA can have on the emotions and feelings of families, not only because it is sensitive to approach them in such an emotionally disturbing period, but also because of the visual impact that CDAs leave on the bodies of their relatives. In spite of this, health professionals still anticipate good acceptance of both methods, if the procedures are adequately explained. Some previous studies have similarly reported that this perceived family reluctance may not necessarily exist [26].

Poor access to family decision makers and people dying at home without entering in contact with the health system are common barriers mentioned both for CDA and MIA. In support to the argument that MIA could be implemented to investigate CoD for deaths occurring outside of the health system, our results revealed two facilitators. First, the MIAs were considered to be *simple* and *easy*, therefore not necessarily requiring highly specialized professionals to conduct them, an attractive concept to the health providers, who labelled this tool as *less specialized*. Second, the consistent perception of the MIAs as *cheaper* and *quicker* than the CDAs was an additional facilitator, not only in the context of implementing them in resource-constrained settings, but also in view of a less disruptive and a more time effective procedure during the moments of grief.

The acceptability results so far are in favour of implementation of the MIA in contribution to a better understanding of the real causes of mortality in resource-constrained settings, should some of the identified perceived limitations be addressed. Understanding the issue from a theoretical perspective, would potentiate even more a systematic approach to address the limitations and enhance the facilitators. Considering that health professionals were approached to discuss MIA by looking at it as an innovation (and particularly taking into account that even without probing in that direction their tendency was almost inevitably to compare it with CDAs), the Diffusion of Innovations Theory (DOI) was deemed appropriate to support the examination of the key issues addressed by the study. Among the four main elements in the DOI (i) the innovation itself, ii) the communication channels; iii) time; and iv) the social system) [13], our focus was on participants' views on the characteristics of the innovation itself (relative advantage; compatibility; complexity; trialability and observability) [13] which were used for describing our findings regarding the MIA.

Regarding its relative advantage, which is the degree to which an innovation is perceived as better than the idea it supersedes [13], the MIA was perceived easier to perform, cheaper, less time consuming, although also less accurate than the CDA. Its reduced invasive nature was a strong additional advantage.

Concerning compatibility, i.e., the degree to which an innovation is perceived as being consistent with the existing values, past experiences, and needs of potential adopters [13], there was no evidence that the MIAs would clash with local practices, and requirements, but timings should be respected. There is a shared perception that it is important to know the CoD and that, from a public health perspective, the implementation of MIAs could strengthen the current understanding of mortality causes. However, caution should be observed not to present the MIA as an audit of clinical practice, but as a support tool for clinicians in LMICs.

The MIA complexity (the degree to which an innovation is perceived as difficult to understand and use) [13] was viewed as minimal or non-existing, as evidenced by the discourse describing it as an easy technique. However, it was acknowledged that implementation might be hindered by the current situation of deaths occurring at home and burials at the community without notification to health authorities.

Trialability, which is the degree to which an innovation may be experimented with on a limited basis [13], was evidenced through the six health providers from the HCM who were performing CDAs routinely when the MIA technique was introduced. Those participants' statements that MIA was quicker and easier were based on their experience with the technique. Through trialability, they maintained their pre-existing concerns regarding the risk of infection of any given post-mortem method.

Observability is the degree to which the results of an innovation are visible to others [13]. In this analysis, observability could be confirmed beyond the context of the health professionals, as MIAs were referred to imply advantages not only for the families, but also for the health system stakeholders and the global health community. In this respect, MIAs have been meteorically embraced for mortality surveillance, even before the tool had been fully validated [29,30].

This study has shown that healthcare providers, both at the formal, but importantly so, also at the informal level, can act as powerful advocates and detractors of the MIAs. Our results reveal that in Southern Mozambique, MIAs seem to be supported and encouraged by this particular professional group, as they seem to value more the associated advantages than any potential drawbacks, and that in their view such drawbacks are addressable at local level. Since healthcare providers may appear naturally suitable to receive training and become in charge of conducting MIAs in the future, and already deal with deceased individuals' relatives in different key moments, it appears critical to foster adequate discussions to continuously inform and involve them in the consent process, the performance of the procedure, and the dissemination of CoD findings. Moreover, training and encouragement on best practices to interact with families and communities more effectively will be needed.

An important limitation of this study is that the data used were collected to answer a broader research question involving a wide range of target groups, from which a sub-sample of healthcare providers' accounts was drawn for this particular analysis. As a qualitative study, the sample size limits generalizability beyond the specific group of healthcare providers serving two very specific areas (Manhiça district and Maputo city), which may also not be representative of the perceptions, views and attitudes in other settings. Finally, post-mortem methods were presented and discussed with study participants from Manhiça as hypothetical scenarios, as MIAs had not been implemented there yet, so there is a need to validate these results under real-life conditions of MIA implementation in the field.

## Conclusions

In the view of Mozambican healthcare providers, the use of post-mortem procedures is compatible with most of their values, being the most important the willingness to know the CoD. Health care providers generally found the MIA more acceptable and feasible than CDAs. MIA is presented less favourably in contrast to the CDA only in terms of its perceived lower accuracy and auditing potential, but it is presented favourably when compared to the CDA for being less complex, cheaper, not disfiguring and for posing less risk of infection among the performers. However, its simplicity could be jeopardized by the lack of support from community leaders, lack of notification systems for deaths at the community, fatalistic view of the death phenomenon, and shortage of equipment and human resources at health facilities. All of the perceived strengths and limitations of MIAS were experienced by some of the participants, confirming the trialability and observability of the MIA as an innovation likely to be adopted.

Healthcare providers represent the most receptive professional group to the concept of post-mortem procedures, and may become key advocates for future use of MIAs in settings where more invasive methods such as the CDA would not be feasible. Nevertheless, it would be important to address their concerns related to disclosure of clinical errors and to ensure comprehensive training on all relevant aspects of the implementation of this innovative technique.
